# Cyclone Freddy in Malawi: Reflections from a primary care perspective

**DOI:** 10.4102/phcfm.v15i1.4142

**Published:** 2023-06-21

**Authors:** Prosper Lutala, Martha Makwero

**Affiliations:** 1 Department of Family Medicine, School of Medicine and Oral Health, Kamuzu University of Health Sciences, Blantyre, Malawi

Cyclones (or tropical storms) have become more and more frequent in the world because of the effects of climate change.^1,2^ The intensity of the cyclone, the country’s socio-economic status and resilience of the health system determine the level of destruction. A cyclone is a trilogy of destruction – intense circular storms, very high continuous winds of more than 119 kilometres per hour, and heavy rains.^3^

Tropical Cyclone Freddy in March 2023 mainly affected the southern part of Malawi.^4^ Flash floods and severe mudslides were the main causes of deaths and destruction of homes, buildings and other infrastructure.^2^ Of note is that Freddy occurred amidst a cholera outbreak, which started almost 2 months prior to the cyclone and had already put strain on the health system. In addition, Freddy was a new experience for most Malawians and the government in terms of its strength, location and consequences. Thus, the government and health system were not prepared for the scale of the devastation.

As of 25 March 2023 the Department of Disaster Management (DoDMa) reported that 126 577 people (563 households) were displaced as a result of winds, floods, and landslides. The death toll had risen to 511, while 533 people were missing, and 1724 were injured. The DoDMa noticed the damage to the water supply system and 918 boreholes were totally submerged, with an estimated 1 008 976 people in urgent need of access to clean water and sanitation.^3^ The government proclaimed a state of emergency on 13 March 2023.^5^ A total of 79 health facilities were affected, with 74 of them functional, but inaccessible, while five had suspended their operations altogether because of a significant disruption of infrastructure, water or electricity supply, as well as medical equipment or supplies.^3^ Most internally displaced people (IDP) were gathered in schools and make-shift camps. Humanitarian aid was mobilised, locally and internationally, and coordinated by DoDMa.

The disruption to lives and livelihoods was huge and included primary health care. Firstly, access to health care was hampered because of destruction of road networks and bridges. The referral hospitals were overwhelmed from the huge demand for healthcare services. There was a reported shortage of medical supplies and inadequate medical personnel to cover the needs. Of note, was the urgent need for maternal and child health services, in the midst of the disaster, yet these were barely accessible. Secondly, IDPs faced a number of health challenges arising from poor sanitation and overcrowding. An escalation of water-borne and respiratory diseases, including cholera, was anticipated, especially among the most vulnerable, such as children and the elderly. Families faced acute hunger as they were already living in poverty and now lost their livelihoods. Furthermore, there was a risk of sexual assaults in the camps for IDPs. The high levels of grief and loss were a recipe for mental health disorders, both for victims and healthcare workers and, the need for mental health services was unmatched. It is therefore evident that these health challenges were overwhelming, especially for a healthcare system that was already battling a severe cholera outbreak.^4,5^

Despite the disruption, life must eventually return to some form of normalcy. Internally displaced people (IDPs) must be decamped, and the country’s systems must recover, rebuild and learn. Such loss and damage as a result of climate change in low-income countries that have not contributed to the problem should be compensated for from high-income countries that are responsible for emissions.^6^ While the needs are enormous, the dependency on external donations must be tampered by local measures, built on deep knowledge of the context and resilience of locals. Malawi must do her homework and implement its own sustainable solutions based on local capacity and resources.

Family medicine as a context-sensitive discipline, addressing local needs through primary health care, has to be in the forefront of building community resilience to future extreme weather events. From a primary health care and family medicine perspective, however, more can be done, such as lobbying for drastic measures in reducing the emissions of carbon dioxide in the atmosphere. Organisations, such as the World Organization of Family Doctors (WONCA), must lobby internationally on the health effects of climate change. A community-orientated primary care approach with a focus on the environmental determinants of health and vulnerabilities at a local level, informed by planetary health, can help to build resilience. This includes the emphasis on community empowerment and multisectoral action. However, the current curriculum in family medicine does not teach such competencies. From experiences, such as Cyclone Freddy, one can see the need to build better curricula that recognise these ecological challenges and help family physicians build more resilient communities.

Leadership skills, the basics of environmental and planetary health, and disaster preparedness and management, are the ingredients that should be added to training programmes. This raises again the need for inter-professional training to deal with these emerging complex issues.^7,8,9^ Training institutions need to invest in curricula that build the next generation of healthcare workers who are able to prepare for and respond to the next disaster.^7^
